# Supplementary material for: A UMLS-based spell checker for natural language processing in vaccine safety
Source: BMC Med Inform Decis Mak. 2007 Feb 12;7:3. doi: 10.1186/1472-6947-7-3 (PMC1805499; doi:10.1186/1472-6947-7-3)
Supplement: Additional file 13 — RAP application directory. Sets up directory for RAP (RDF application for PHP) source code [file 1472-6947-7-3-S13.gz › rap/doc/phpdoc/utility/RDFUtil.html]

xml version="1.0" encoding="iso-8859-1"?


Docs For Class RDFUtil


## Class RDFUtil

Description

Description |
Methods (details)

Useful utility methods.

Static class.

History:

- 03-29-2005 : Function guessNamespace() and visualizeGraph() added (anton1@koestlbacher.de)
- 12-06-2004 : improved namespace handling in function
- 09-10-2004 : added support for OWL and infered statements
- 11-18-2003 : Function writeAsHtmlTable() htmlspecialchars & nl2br
  for displaying literals added.
- 04-23-2003 : Chunk\_split() removed from writeHTMLTable
- 12-04-2002 : Added support for "rdf:datatype" in writeHTMLTable
- 10-03-2002 : Green coloring for RDF\_SCHEMA nodes added to writeHTMLTable
- 09-10-2002 : First version of this class.

- access: public
- author: Chris Bizer <chris@bizer.de>, Daniel Westphal <dawe@gmx.de>
- author: Anton K�stlbacher <anton1@koestlbacher.de>
- version: V0.9.1

Located in /util/RdfUtil.php (line 30)

```
Object
   |
   --RDFUtil
```

Method Summary

Description |
Methods (details)

object Resource
createOrd
(Integer $num)

String
escapeValue
(String $textValue)

string
getLocalName
(Object $resource)

string
getNamespace
(Object $resource)

Integer
getOrd
(object Resource $resource)

void
getPrefix
(String $uri)

string
guessName
(String $uri)

string
guessNamespace
(String $uri)

string,
guessPrefix
(string $uri, object &$model)

boolean
isRDF
(Object $resource)

string,
visualizeGraph
(object Model &$model, [string $format = "input\_dot"], [boolean $short\_prefix = TRUE])

void
writeHTMLTable
(object MemModel &$model)

Methods

Description |
Methods (details)

createOrd (line 175)

Creates ordinal RDF resource out of an integer.

- access: public

object Resource

createOrd
(Integer $num)

- Integer
  $num

escapeValue (line 140)

Escapes < > and &

- access: public

String

escapeValue
(String $textValue)

- String
  $textValue

getLocalName (line 73)

Delivers the Localname (without the namespace prefix) out of the URI of a Resource.

- access: public

string

getLocalName
(Object $resource)

- Object
  $resource: Resource $resource

getNamespace (line 62)

Extracts the namespace prefix out of the URI of a Resource.

- access: public

string

getNamespace
(Object $resource)

- Object
  $resource: Resource $resource

getOrd (line 157)

Converts an ordinal RDF resource to an integer.

e.g. Resource(RDF:\_1) => 1

- access: public

Integer

getOrd
(object Resource $resource)

- object Resource
  $resource

getPrefix (line 100)

Short Prefix for known Namespaces by given URI

- access: public

void

getPrefix
(String $uri)

- String
  $uri

guessName (line 51)

Delivers the name out of the URI (without the namespace prefix).

- access: public

string

guessName
(String $uri)

- String
  $uri

guessNamespace (line 39)

Extracts the namespace prefix out of a URI.

- access: public

string

guessNamespace
(String $uri)

- String
  $uri

guessPrefix (line 350)

Short Prefix for known and/or parsed Namespaces by given URI and Model Uses $default\_prefixes defined in constants.php and getParsedNamespaces() Returns FALSE if no matching prefix is found

- return: boolean
- throws: PhpError
- access: public
- author: Anton K�stlbacher <anton1@koestlbacher.de>

string,

guessPrefix
(string $uri, object &$model)

- string
  $uri
- object
  $model

isRDF (line 129)

Tests if the URI of a resource belongs to the RDF syntax/model namespace.

- access: public

boolean

isRDF
(Object $resource)

- Object
  $resource: Resource $resource

visualizeGraph (line 395)

Generates a dot-file for drawing graphical output with the

graphviz-application which can be downloaded at http://www.graphviz.org If the graphviz-application is installed and its path is set to the correct value in constants.php we can directly generate any file format graphviz supports, e.g. SVG, PNG Parameters: model to visualize, output format, use prefixes

!!!!!!!!!!!!!!!!!!!!!!!!!!!!!!!!!!!!!!!!!!!!!!!!!!!!!!!!!!!!!!!!!!!!!! WARNING: Graphviz can be slow with large models. !!!!!!!!!!!!!!!!!!!!!!!!!!!!!!!!!!!!!!!!!!!!!!!!!!!!!!!!!!!!!!!!!!!!!!

- return: binary
- throws: PhpError
- access: public
- author: Anton K�stlbacher <anton1@koestlbacher.de>

string,

visualizeGraph
(object Model &$model, [string $format = "input\_dot"], [boolean $short\_prefix = TRUE])

- string
  $format
- boolean
  $short\_prefix
- object Model
  &$model

writeHTMLTable (line 186)

Prints a MemModel as HTML table.

You can change the colors in the configuration file.

- access: public

void

writeHTMLTable
(object MemModel &$model)

- object MemModel
  &$model

#### Inherited Methods


Inherited From Object

> Object::toString()

Documentation generated on Thu, 7 Jul 2005 13:43:09 +0200 by phpDocumentor 1.3.0RC3
